# Equilibrium crystal shape of GaAs and InAs considering surface vibration and new (111)B reconstruction: ab-initio thermodynamics

**DOI:** 10.1038/s41598-018-37910-y

**Published:** 2019-02-04

**Authors:** In Won Yeu, Gyuseung Han, Jaehong Park, Cheol Seong Hwang, Jung-Hae Choi

**Affiliations:** 1 Center for Electronic Materials, Korea Institute of Science and Technology, Seoul, 02792 Korea; 2 Department of Materials Science and Engineering and Inter-University Semiconductor Research Center, Seoul National University, Seoul, 08826 Korea

## Abstract

This work reports on the theoretical equilibrium crystal shapes of GaAs and InAs as a function of temperature and pressure, taking into account the contribution of the surface vibration, using ab-initio thermodynamic calculations. For this purpose, new (111)B reconstructions, which are energetically stable at a high temperature, are suggested. It was found that there was a feasible correspondence between the calculated equilibrium shapes and the experimental shapes, which implied that the previous experimental growth was performed under conditions that were close to equilibrium. In this study, GaAs and InAs were selected as prototype compound semiconductors, but the developed calculation methodology can also be applied to other III–V compound semiconductor materials.

## Introduction

The integration of the III–V compounds on a Si substrate was recently studied to exploit the higher electronic performance of III–V compound semiconductors on Si wafer^1–3^. There are, however, several technical difficulties to overcome, such as dislocations induced by the lattice mismatch, the cracks due to the differences in the thermal expansion coefficient, and the anti-phase boundary resulting from the growth of the polar III–V compounds on nonpolar Si. Many attempts have been made to solve these problems, and it has been found that selective area growth (SAG) is one of the effective ways of suppressing the propagation of dislocations and cracks as well as lowering the density of the anti-phase boundary^4–7^. The III–V compounds grown via SAG is in the form of quantum dots^6,8–16^ or nanowires^10,11,17^. Their crystal morphology changes depending on the temperature (T) and pressure (P) of growth conditions^10,11,18^. Therefore, many experiments have been carried out to tailor the crystal morphology by controlling T and P to obtain the optimum device characteristics. Despite the crucial role of the surface energy and its anisotropy on the crystal morphology, a way of theoretically predicting both the surface energy and the crystal shape as a function of T and P has yet to be developed^8,19^.

Previous density functional theory (DFT) calculations reported the equilibrium crystal shape (ECS) of the III–V compounds as a function of chemical potential^20–22^. While such an approach can show the tendency of the ECS under either the III- or V-rich condition, it is necessary to calculate the ECS as a function of the practical experimental variables (thermodynamic parameters) of T and P to directly compare the calculated ECSs with the experimentally grown shapes. In this study, therefore, the ECSs were predicted as a function of T and P from the surface energies calculated for the given T and P values using the ab-initio thermodynamic methodology proposed by the authors elsewhere^23^. For this purpose, GaAs and InAs were selected as the prototype III–V compound semiconductors.

To obtain the ECS, the following steps were carried out. The first step was calculating the surface energies of several low-index facets as a function of T and P using ab-initio thermodynamics, and constructing the Wulff shape based on the calculated surface energy values^24,25^. We showed that the typical experimental growth conditions correspond to the III-rich condition rather than the V-rich condition. The second step was considering the influence of the surface vibration on the surface energy and the ECS, which had not been calculated for the III–V compounds^20–22^. This study systematically calculated the surface energies of the low-index facets in the III–V compounds considering the surface vibration. The last step was suggesting and identifying the new surface reconstructions for the (111)B facets, III vacancy(2 × 2) in GaAs and InAs, because the (111)B facets were usually shown in the experimentally grown materials^10–13^. Nonetheless, the previous theoretical works predicted that the ECS does not contain the (111)B facets in the III-rich condition^20–22^. At each step, the calculation results were consistently compared with those in the previous experimental works on GaAs and InAs. At the last of the aforementioned steps, the correspondence between the calculated ECS and the experimentally grown shapes was elucidated, suggesting that the practical experimental conditions were close to the equilibrium conditions. This work clearly shows the importance of the surface vibration in the ECS, and the possibility of the existence of new (111)B reconstructions. The general methodology developed in this work can be applied to other III–V materials.

## Results and Discussion

**Figure 1:**
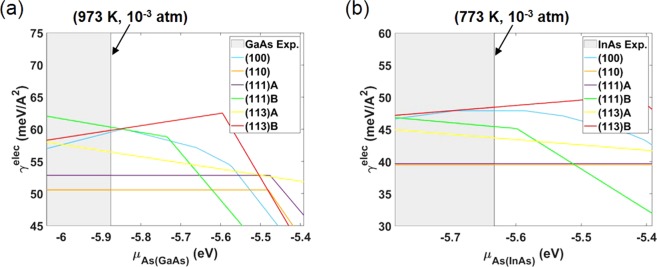
Calculated minimum surface energies of (**a**) GaAs and (**b**) InAs for each surface orientation. The shaded area corresponds to the experimental growth conditions.

The minimum surface energies for several low-index surfaces were obtained by calculating the surface energies of various reconstructions for each surface. First, all the reported reconstructions for (100)^20,23,26^, (110)^20^, (111)A^20,27^, (111)B^20,27,28^, (113)A^29–31^, and (113)B^29,31,32^ were identified (Supplementary Fig. S1). Then the electronic surface energy (*γ*^*elec*^) of each reconstruction was calculated as a function of *μ*_*As*(*GaAs*)_ for GaAs (Supplementary Fig. S2) and for InAs (Supplementary Fig. S3). At each chemical potential, the surface energy of each surface was determined by reconstructing the atomic structure of the corresponding surfaces so that they would have the minimum surface energy. Figure 1 shows the minimum surface energies of the low-index surfaces. In this figure, the point where the slope of the line changes corresponds to the transition point of the reconstruction of each surface. By combining the equilibrium condition of equation (7) and the calculated chemical potential of As gas (*μ*_*As*(*Gas*)_) as a function of T and *P*_*As*_, the experimental growth conditions can be denoted as the shaded area for GaAs^6,8–11,17–19^ in Fig. 1(a) and for InAs^12,13^ in Fig. 1(b). The line of the highest value of *μ*_*As*(*GaAs*)_ and *μ*_*As*(*InAs*)_ among the experimental growth conditions is indicated (Fig. 1). It should be pointed out that the typical experimental growth conditions of both GaAs and InAs fall under the III-rich condition. The minimum surface energies in each surface orientation are presented as a function of T and *P*_*As*_ in the range of experiment conditions using the equilibrium condition of equation (7) (Supplementary Fig. S4(a,b)). Generally, the *γ*^*elec*^ for InAs is approximately 10 meV/Å^2^ lower than that for GaAs due to the weaker bonding between In and As than between Ga and As.

**Figure 2:**
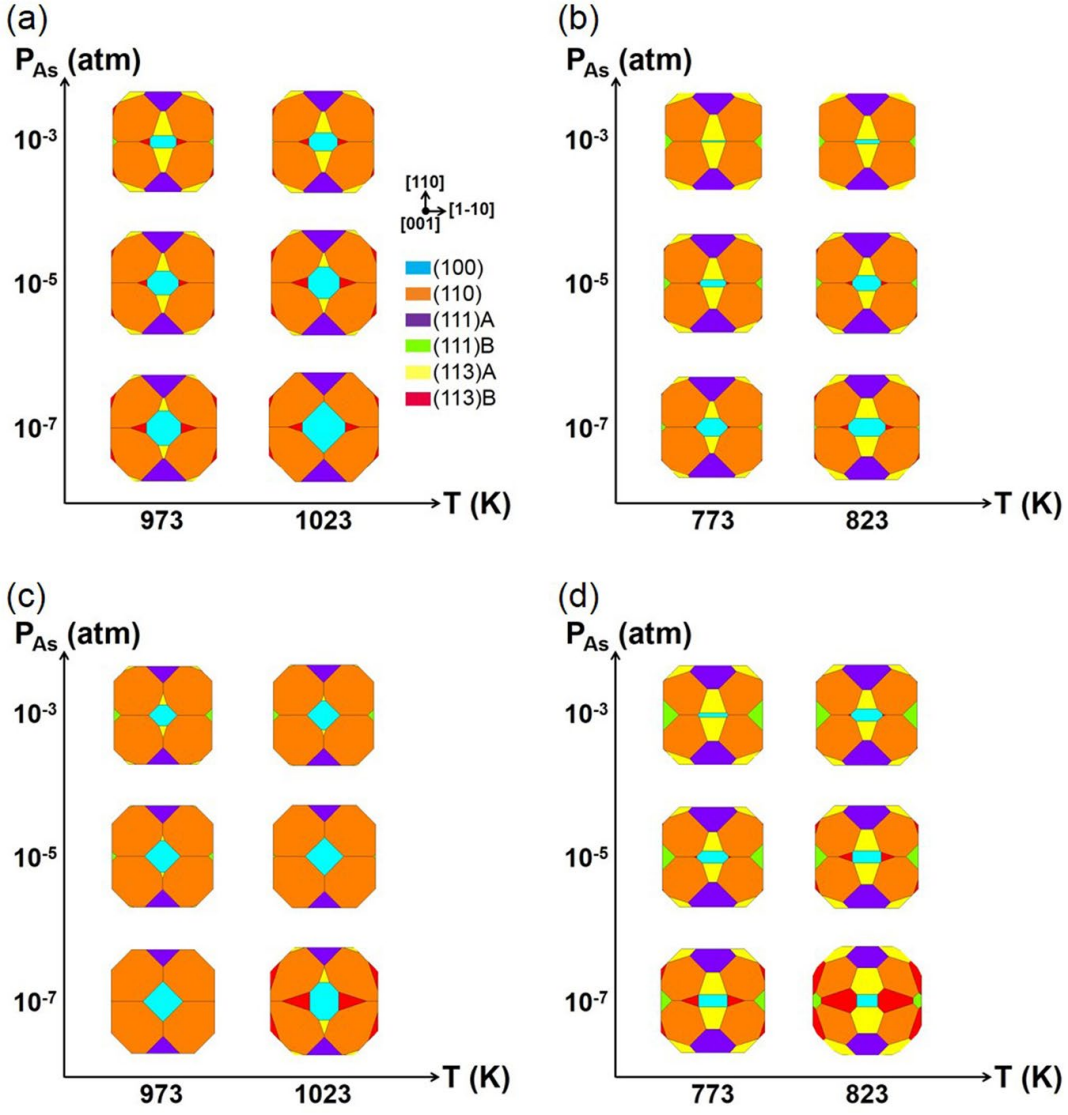
Top view of the ECSs of (**a**) GaAs and (**b**) InAs along the [001] direction at around the experimental growth conditions. ECSs of (**c**) GaAs and (**d**) InAs after considering the surface vibration.

From the calculated *γ*^*elec*^(T, *P*_*As*_) for each surface orientation, the ECS as a function of T and *P*_*As*_ can be obtained through Wulff construction^24,25^. Figure 2(a,b) show the calculated ECSs of GaAs and InAs, respectively, in the range of T and *P*_*As*_ corresponding to the shaded areas in Fig. 1(a,b). They are mainly composed of (100), (110), (111)A, and (113)A facets, with a small portion composed of (113)B facets and with a negligible area of (111)B facets. In contrast, the experimentally grown shapes of GaAs^10,11^ are composed of (100), (110), (111)A, and (111)B facets showing similar surface areas of (111)A and (111)B, while those of InAs^12,13^ are composed of (100), (110), (111)A, and (111)B facets with several other unidentified high-index surfaces. In fact, previous theoretical works also described the absence of the (111)B facets in the III-rich condition^20–22^. They suggested that it could be attributed to the omission of the high T reconstruction of the (111)B surface, such as ($\sqrt{19}\times \sqrt{19}$), which was beyond the computational capability at the time that such theoretical works were conducted^20–22^. Although this study calculated the electronic surface energies (*γ*^*elec*^) of more reconstructions compared to the previous works, including (111)B ($\sqrt{19}\times \sqrt{19}$), (113)A, and (113)B (Supplementary Figs S2 and S3), the discrepancy between the calculations and the experiments remains unaddressed. Therefore, surface vibration was additionally considered, as discussed below.

Surface vibration has been reported to have a significant influence on the surface phase diagrams of ZnO(0001)^33^ as well as the electronic properties, such as the metal-insulator transition in the Si(111)-In nanowire^34^. For the prediction of the ECS, the surface vibration may be more crucial because its effects on the various surfaces are anisotropic, resulting from the significantly different bonding geometry for each reconstruction (Supplementary Fig. S1). To consider the effects of surface vibration on the ECS, surface energies composed of the electronic and vibrational terms (*γ* = *γ*^*elec*^ + Δ*γ*^*vib*^) were calculated (Supplementary Fig. S4(c,d)) for several reconstructions with low electronic surface energy (the bold lines in Supplementary Figs S2 and S3). When the surface vibration is considered, the surface energies decrease by ~5 meV/Å^2^. The surface vibration of only the atoms at the uppermost to the 3^rd^ layer was considered for each reconstruction. This is because the bonding geometries and vibration of the atoms situated deeper than the 3^rd^ layer from the uppermost layer are almost identical with those of the atoms in the bulk state (Supplementary Fig. S5).

Using the *γ*(T, *P*_*As*_) value obtained through calculation in each surface orientation, the ECSs(T, *P*_*As*_) considering the surface vibration were obtained for GaAs in Fig. 2(c) and for InAs in Fig. 2(d). Their differences from those in Fig. 2(a,b) indicate that the effects of the surface vibration on the surface energy (Δ*γ*^*vib*^) depend on the reconstruction, surface orientation, and materials. This is because the bonding geometry varies with the surface reconstruction and orientation, and the surface vibration varies with the atomic mass. The surface vibration shrinks the surface area of (113)A facets for the ECS of GaAs, which agrees better with the experimental results^10,11^. On the other hand, the surface vibration enlarges the (113)A and (113)B facets for the ECS of InAs, which is an indication of the several unidentified high-index surfaces in the experiments^12,13^. Through these intensive calculations of the ECS, the various reconstructions, including (111)B ($\sqrt{19}\times \sqrt{19}$), (113)A, and (113)B, as well as the surface vibration, were systematically considered. The (111)B facets, however, were still identified to be energetically unfavorable in both GaAs and InAs.

**Figure 3:**
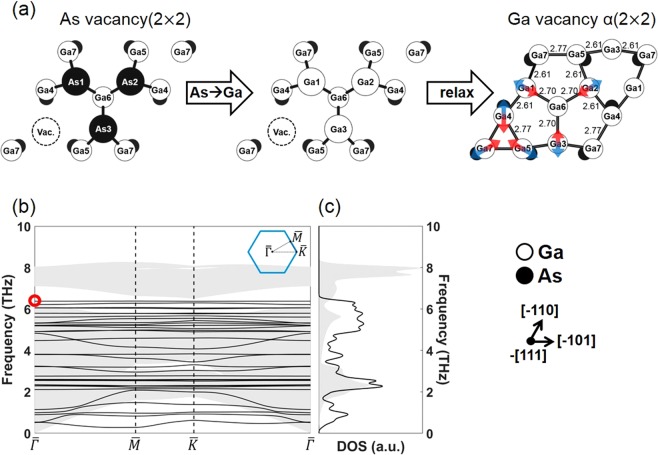
(**a**) Atomic structure of (111)B Ga vacancy α(2 × 2) for GaAs. The arrows denote the surface phonon mode with the highest frequency at the $\bar{{\Gamma }}$ point for the atoms at the uppermost layer. (**b**) Band structure and (**c**) DOS of (111)B Ga vacancy α(2 × 2) (black lines), with the projection of bulk phonon dispersion onto the 2D Brillouin zone (shaded areas).

In this study, therefore, a new high T reconstruction of the (111)B surface, III vacancy(2 × 2), was suggested. This reconstruction is made from the substitution of the uppermost As atoms by the group III atoms in (111)B As vacancy(2 × 2), as shown in Fig. 3(a). The introduction of this new reconstruction is based on the fact that there is a general tendency for a reconstruction with more III atoms than V atoms on its surface to be stable in the III-rich condition, and vice versa^23^. The structural stability of this new reconstruction in GaAs, (111)B Ga vacancy α(2 × 2), was confirmed by the absence of any negative frequency in the band structure (Fig. 3(b)) and density of states (DOS) (Fig. 3(c)) for the surface phonon. It was noted that the frequency of the surface phonon was significantly lowered compared to the frequency of the bulk GaAs phonon, presumably due to the weaker bonding between Ga atoms than between Ga and As atoms. After full relaxation, the height of the Ga atoms at the uppermost layer became identical to that of the Ga atoms at the 2^nd^ layer from the uppermost layer, and the bond lengths between the Ga atoms at the surface, denoted in Fig. 3(a), became almost identical to the bond lengths of the orthorhombic bulk Ga (2.45–2.73 Å) calculated under the same calculation conditions. The surface phonon mode at the $\bar{{\Gamma }}$ point with the highest frequency (the red circle in Fig. 3(b), 6.38 THz) is denoted by arrows for the atoms at the uppermost layer.

**Figure 4:**
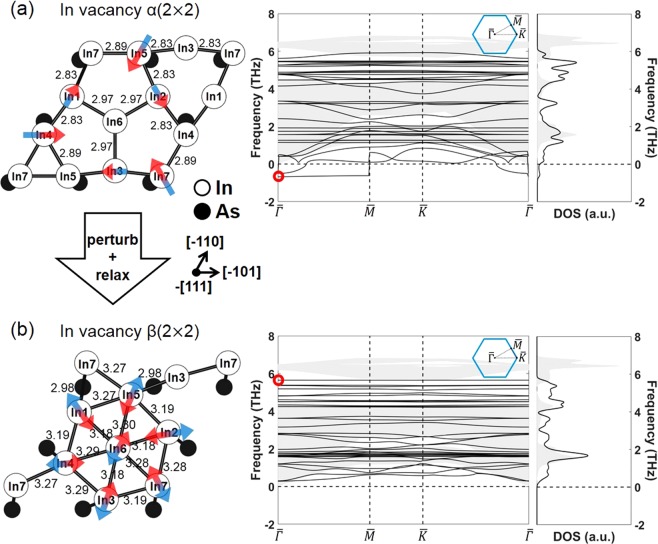
Atomic structures, band structures, and DOSs of the surface phonon for the (**a**) (111)B In vacancy α(2 × 2) and (**b**) (111)B In vacancy β(2 × 2) in InAs. The arrows denote the surface phonon mode at the $\bar{{\Gamma }}$ point with (**a**) a negative frequency and (**b**) the highest frequency for the atoms at the uppermost layer. The projection of bulk phonon dispersion onto the 2D Brillouin zone is also shown as a shaded area.

The stability of InAs(111)B In vacancy α(2 × 2), whose structure is the same as that of GaAs(111)B Ga vacancy α(2 × 2), was also investigated by examining the band structure and DOS of the surface phonon (Fig. 4(a)). In contrast to the case of GaAs, this reconstruction shows a negative frequency of the surface phonon even after the relaxation calculation was converged, indicating that the structure is metastable and the restoring force does not exist for the movement along the negative phonon mode. The surface phonon mode at the $\bar{{\Gamma }}$ point with a negative frequency (red circle) is indicated by arrows for the atoms at the uppermost layer. To propose another stable reconstruction, a relaxation calculation was performed after perturbing the uppermost atoms along the direction of the arrows. A new reconstruction without any negative frequency in the surface phonon was obtained (Fig. 4(b)) and it was denoted as (111)B In vacancy β(2 × 2). Note that the two structures in Fig. 4(a,b) show different bond lengths, and that the bond length of (111)B In vacancy β(2 × 2) is almost identical to those of the tetragonal bulk In (3.22–3.29 Å) calculated under the same DFT conditions. The frequency of the surface phonon in (111)B In vacancy β(2 × 2) is also substantially lower compared to the frequency of the bulk InAs phonon. The surface phonon mode of this reconstruction at the $\bar{{\Gamma }}$ point with the highest frequency (5.65 THz) is denoted by arrows for the atoms at the uppermost layer. In addition, it was noted that the phonon frequencies of the surface and bulk states for InAs are much lower than those for GaAs due to the weaker bonding and higher mass of InAs than GaAs.

**Figure 5:**
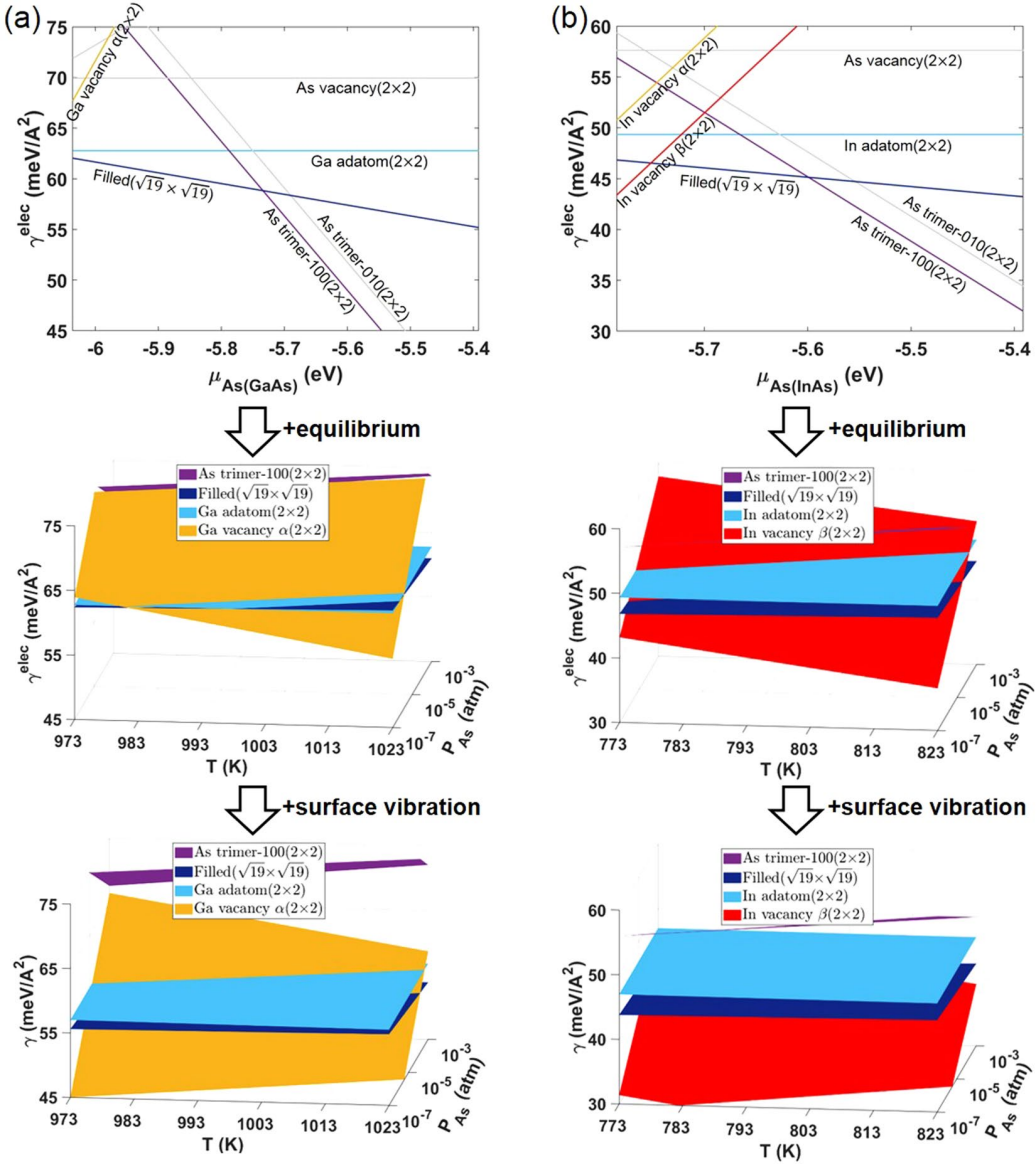
Calculated surface energies of the (**a**) GaAs(111)B and (**b**) InAs(111)B reconstructions, including III vacancy(2 × 2), and the surface vibration effects on each reconstruction. Note that the top figure in (**a**) is identical to Supplementary Fig. S2(d), except the Ga vacancy α(2 × 2) reconstruction, and that the top figure in (**b**) is identical to Supplementary Fig. S3(d), except the In vacancy α and β(2 × 2) reconstructions.

In addition to the structural stability, the energetic stability of the new (111)B III vacancy(2 × 2) reconstructions were confirmed by calculating the surface energy. Figure 5(a) shows the surface energies of the GaAs(111)B reconstructions, including (111)B Ga vacancy α(2 × 2) (different from Supplementary Fig. S2(d), which does not have (111)B Ga vacancy α(2 × 2)). Likewise, Fig. 5(b) shows the surface energies of the InAs(111)B reconstructions, including (111)B In vacancy α(2 × 2) and In vacancy β(2 × 2) (different from Supplementary Fig. S3(d), which does not have (111)B In vacancy α and β(2 × 2)). (111)B III vacancy(2 × 2) is less stable than (111)B ($\sqrt{19}\times \sqrt{19}$) with regard to the electronic surface energy (*γ*^*elec*^). In constrast, the absolute reduction of surface energy by the surface vibration (|Δ*γ*^*vib*^|) of the (111)B Ga vacancy α(2 × 2) in GaAs and the (111)B In vacancy β(2 × 2) in InAs is drastically larger than that of the other (111)B reconstructions, which confirms that (111)B III vacancy(2 × 2) is energetically stable in the T and *P*_*As*_ range of interest. This is presumably due to the low frequency of surface vibration resulting from the weak bonding between the group III atoms. The tendency for the surface structure with electronically weak bonding to become more stable in a high T condition due to the low frequency of surface vibration was also reported in the previous calculation for a different material system^34^. These calculation results are consistent with those in the previous experimental studies obtained through electron diffraction^35^ and scanning tunneling microscopy (STM)^36^, suggesting the possibility of surface transition from ($\sqrt{19}\times \sqrt{19}$) to another higher T reconstruction in the (111)B surface.

**Figure 6:**
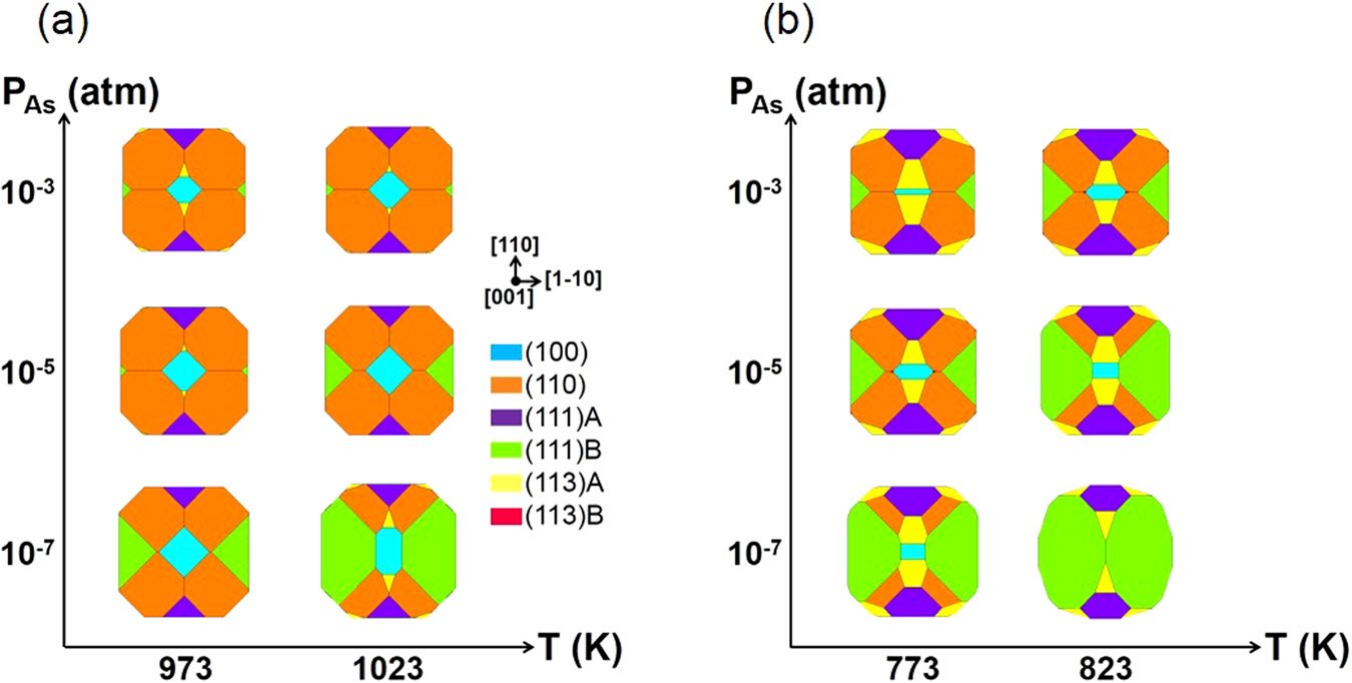
Top view of the ECSs of (**a**) GaAs and (**b**) InAs along the [001] direction at around the experimental growth conditions after including the surface vibration and the newly suggested (111)B III vacancy(2 × 2).

Finally, the ECSs(T, *P*_*As*_) in the T and *P*_*As*_ range of experimental conditions, including both the surface vibration and the new (111)B III vacancy(2 × 2), are shown in Fig. 6. The large reduction of the surface energy of (111)B by the new reconstruction contributes to the obvious existence of the (111)B facets for both GaAs (Fig. 6(a)) and InAs (Fig. 6(b)). The existence of (111)B facets in the ECS calculation of the III–V compounds is shown in the III-rich condition, and this result agrees well with the usual experimentally grown shapes of GaAs^10,11^ and InAs^12,13^. The correspondence between the calculated ECS and the experimental SAG shapes confirms that the experimental growth usually occurred under the conditions near equilibrium.

## Conclusion

The equilibrium crystal shapes (ECSs) of GaAs and InAs were predicted as a function of temperature (T) and pressure (P) using ab-initio thermodynamics. The surface energies of all the reported reconstructions on the (100), (110), (111)A, (111)B, (113)A, and (113)B surfaces were calculated. For this purpose, the surface vibration, which has been ignored in the previous ECS calculations, was taken into account, and it was found to significantly depend on the surface reconstruction, surface orientation, and material type. As a result, the ECS considering the surface vibration was determined to be different from the ECS without considering the surface vibration. In addition, new (111)B III vacancy(2 × 2) reconstructions were suggested, and their stability was confirmed by the surface energy and surface phonon dispersion. Especially, the energetic stability of these reconstructions was confirmed at a high T condition. This was due to the larger surface energy reduction than in the other reconstructions considered resulting from the low frequency of surface vibration. Considering both the surface vibration and the (111)B III vacancy(2 × 2) reconstruction, a correspondence between the calculated ECS and the experiment results could be demonstrated for GaAs and InAs. This correspondence suggests that the previous experimental growth occurred near equilibrium conditions.

## Calculation Methods

Vienna Ab-Initio Simulation Package (VASP)^37–40^ was used for all the DFT calculations, using the projector-augmented wave (PAW)^41,42^ method. Local density approximation (LDA) parameterized by Ceperley^43,44^ was used for the exchange-correlation functional, with the plane wave basis function within 500 eV cutoff energy in all calculations. The 3d, 4 s, and 4p orbitals of Ga; the 4d, 5 s, and 5p orbitals of In; and the 4 s and 4p orbitals of As were treated as valence electrons. For the conventional bulk unitcell, the atomic positions were relaxed until the Hellmann-Feynman forces were less than 0.0001 eV/Å using 12 × 12 × 12 k-points. After generating the slab structures consisting of at least eight atomic layers with more than 10 Å vacuum layer from the relaxed bulk, only the atoms at the top five layers were relaxed until the forces were less than 0.02 eV/Å. The k-points along the in-plane directions for the slab calculations were scaled according to the cell size to keep the same k-point density as bulk. For all slab calculations, the dipole correction along the vacuum direction was considered.

The surface energy (*γ*) of GaAs was calculated based on the energy difference between the surface and the bulk, using the following equation:

1 $$\gamma =\frac{({E}_{surf}^{elec}+{F}_{surf}^{vib})-{N}_{Ga}({E}_{Ga(GaAs)}^{elec}+{F}_{Ga(GaAs)}^{vib})-{N}_{As}({E}_{As(GaAs)}^{elec}+{F}_{As(GaAs)}^{vib})}{A},$$

where ${E}_{surf}^{elec}$, ${E}_{Ga(GaAs)}^{elec}$, and ${E}_{As(GaAs)}^{elec}$ are the electronic ground state energies of the surface slab structure, the Ga in the bulk GaAs, and the As in the bulk GaAs, respectively; ${F}_{surf}^{vib}$, ${F}_{Ga(GaAs)}^{vib}$, and ${F}_{As(GaAs)}^{vib}$ are the vibrational energies of the surface slab structure, the Ga in the bulk GaAs, and the As in the bulk GaAs, respectively; and *N*_*Ga*_, *N*_*As*_, and *A* are the numbers of Ga and As atoms and the surface area of the slab structure, respectively. When the following equilibrium condition of GaAs is satisfied:

2 $$({E}_{Ga(GaAs)}^{elec}+{F}_{Ga(GaAs)}^{vib})+({E}_{As(GaAs)}^{elec}+{F}_{As(GaAs)}^{vib})=({E}_{GaAs(bulk)}^{elec}+{F}_{GaAs(bulk)}^{vib}),$$

$\mathrm{the}\,{E}_{Ga(GaAs)}^{elec}$ and ${F}_{Ga(GaAs)}^{vib}$ terms in equation (1) can be substituted by the electronic ground state energy of the bulk GaAs (${E}_{GaAs(bulk)}^{elec}$) and the vibrational energy of the bulk GaAs (${F}_{GaAs(bulk)}^{vib}$), respectively. Therefore,

3 $$\gamma =\frac{({E}_{surf}^{elec}+{F}_{surf}^{vib})-{N}_{Ga}({E}_{GaAs(bulk)}^{elec}+{F}_{GaAs(bulk)}^{vib})-({N}_{As}-{N}_{Ga})({E}_{As(GaAs)}^{elec}+{F}_{As(GaAs)}^{vib})\,}{A}.$$

Note that the energy of the bulk GaAs can be more easily calculated than that of the Ga in the bulk GaAs. If the electronic energy and vibrational energy terms are separated from each other,

4 $$\gamma ={\gamma }^{elec}+{\rm{\Delta }}{\gamma }^{vib},$$

where *γ*^*elec*^ indicates the calculated surface energy containing only the difference in electronic energy between the surface and the bulk, as follows:

5 $${\gamma }^{elec}=\frac{({E}_{surf}^{elec})-{N}_{Ga}({E}_{GaAs(bulk)}^{elec})-({N}_{As}-{N}_{Ga})({E}_{As(GaAs)}^{elec})\,}{A},$$

and Δ*γ*^*vib*^ is the difference in vibrational energy between the surface and the bulk, as follows:

6 $${\rm{\Delta }}{\gamma }^{vib}=\frac{({F}_{surf}^{vib})-{N}_{Ga}({F}_{GaAs(bulk)}^{vib})-({N}_{As}-{N}_{Ga})({F}_{As(GaAs)}^{vib})\,}{A}.$$

The calculated *γ*^*elec*^ is usually obtained as a function of chemical potential, *μ*_*As*(*GaAs*)_. To calculate the surface energy as a function of T and P, a thermodynamic equilibrium between the surface and the surrounding gas phase was assumed, as shown in the equation below.

7 $${\mu }_{As(GaAs)}={\mu }_{As(Gas)}$$

The gas phase surrounding the surface of GaAs was considered a mixture of As_2_ and As_4_ molecules. The Ga atoms (and the In atoms in the InAs system) were not considered components of the gas phase because the partial pressure of the group III gases is usually over 10 times lower than that of the group V gases in the practical growth conditions for III–V crystals^6,8–13,17–19^. The chemical potential of the As gas phase, *μ*_*As*(*Gas*)_, was calculated as a function of T and P using DFT calculations. The detailed DFT methods of calculating the electronic surface energy (*γ*^*elec*^), chemical potential of gas (*μ*_*As*(*Gas*)_), and vibrational energy (*F*^*vib*^) were described in the authors’ previous study^23^. For InAs, equations (1–7) were also used, with the relevant terms for InAs. For the polar surfaces such as (111)A, (111)B, (113)A, and (113)B, the surface energies were calculated using the wedge-shaped geometry^45^.

## Supplementary information

Supplementary Information: Equilibrium crystal shape of GaAs and InAs considering surface vibration and new (111)B reconstruction: ab-initio thermodynamics
