# Supplementary material for: Equilibrium crystal shape of GaAs and InAs considering surface vibration and new (111)B reconstruction: ab-initio thermodynamics
Source: Sci Rep. 2019 Feb 4;9:1127. doi: 10.1038/s41598-018-37910-y (PMC6361998; doi:10.1038/s41598-018-37910-y)
Supplement: Supplementary file 1 — Supplementary Information: Equilibrium crystal shape of GaAs and InAs considering surface vibration and new (111)B reconstruction: ab-initio thermodynamics [file 41598_2018_37910_MOESM1_ESM.pdf]

Supplementary Information:

Equilibrium crystal shape of GaAs and InAs considering surface vibration and new (111)B reconstruction: ab-initio thermodynamics

In Won Yeu[a,b], Gyuseung Han[a,b], Jaehong Park[a,b], Cheol Seong Hwang[b], and Jung-Hae Choi[a,*]

[a]Center for Electronic Materials, Korea Institute of Science and Technology, Seoul 02792, Korea

[b]Department of Materials Science and Engineering and Inter-University Semiconductor Research Center, Seoul National University, Seoul 08826, Korea

[*]Corresponding author. Tel.: +82 2 958 5488; Fax: +82 2 958 6658

E-mail address: choijh@kist.re.kr (J.-H. Choi)

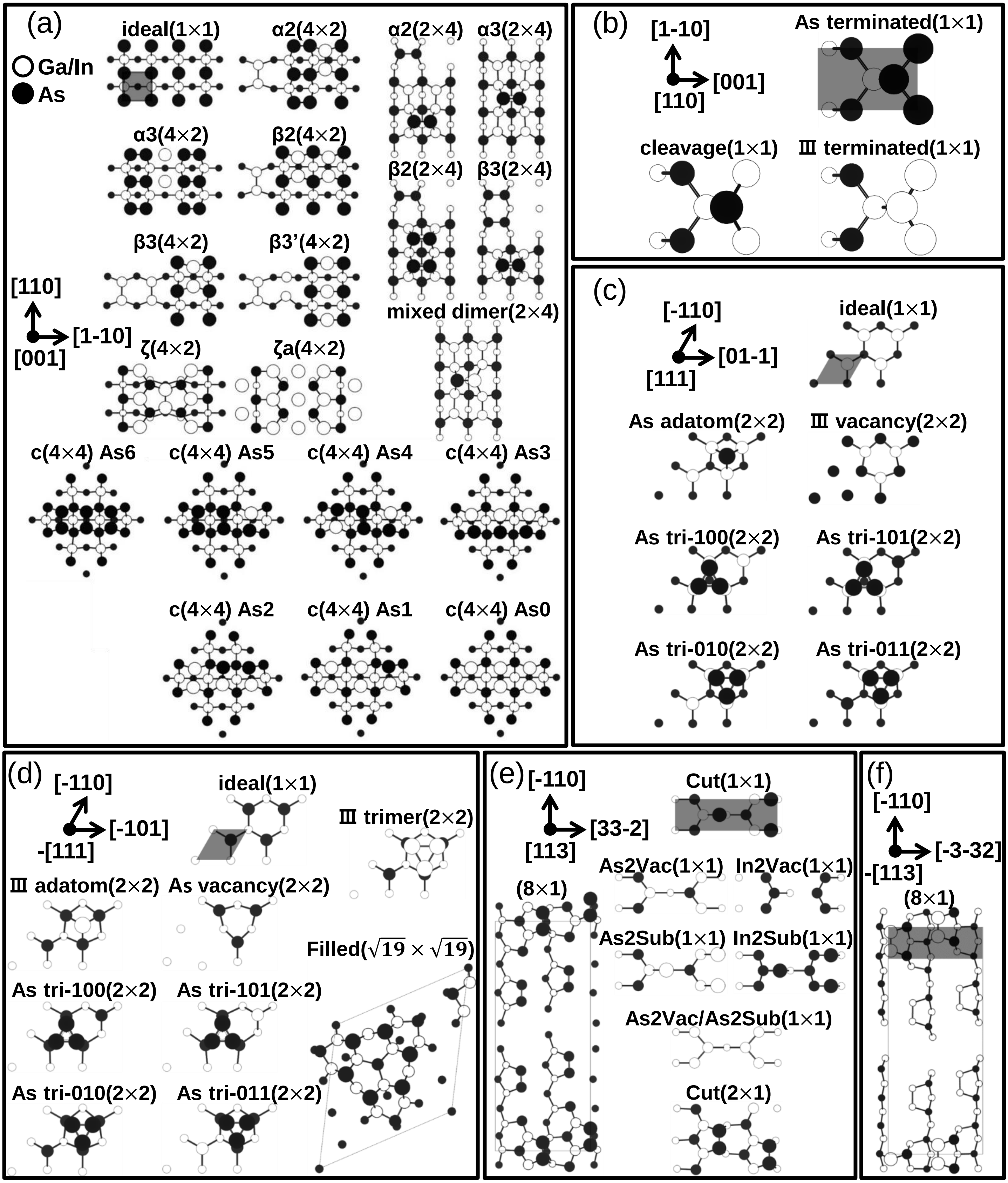

Figure S1. Top view of the calculated atomic structures of the surface reconstructions of the (a) (100), (b) (110), (c) (111)A, (d) (111)B, (e) (113)A, and (f) (113)B surfaces. All the surface areas correspond to the surface unit cell, except the ideal(1×1), whose surface unit cell sizes are indicated by shaded areas. The largest circles indicate the atoms at the topmost layer.

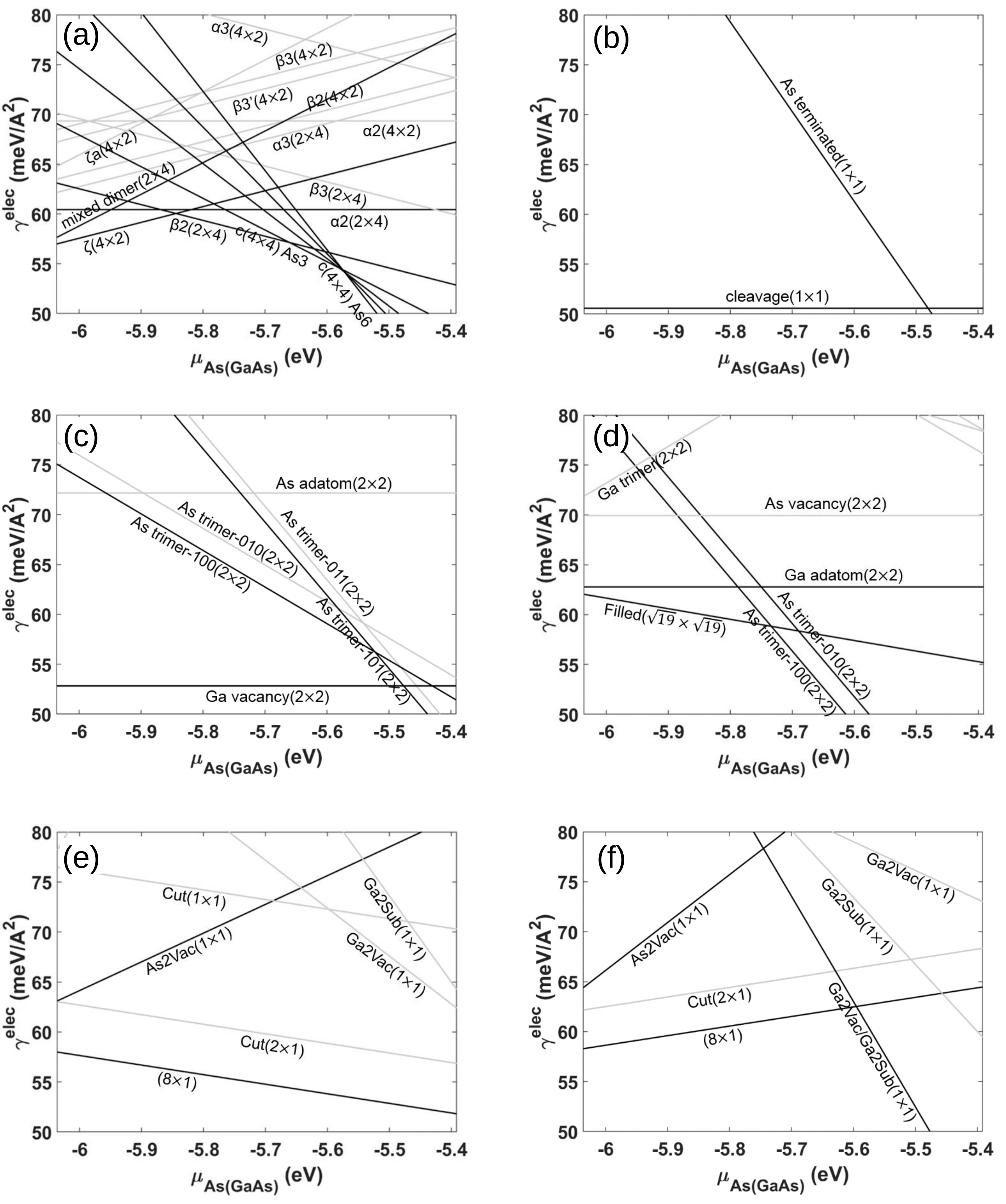

Figure S2. Calculated electronic surface energies of GaAs (a) (100), (b) (110), (c) (111)A, (d) (111)B, (e) (113)A, and (f) (113)B. When the vibrational energy was calculated, only the reconstructions represented by bold lines were considered. Note that Fig. S2(d) is identical to Fig. 5(a), except that there is no Ga vacancy α(2×2).

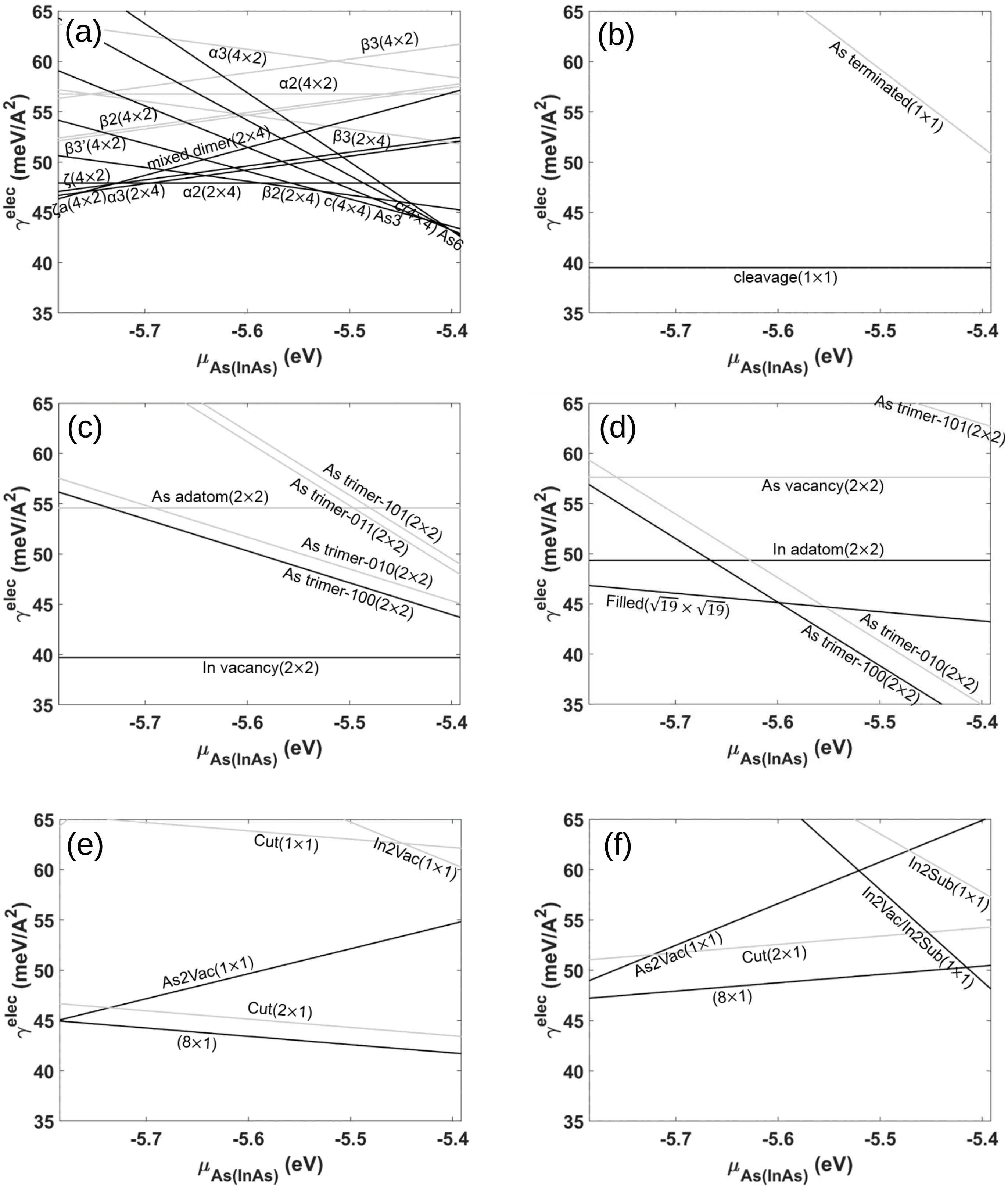

Figure S3. Calculated electronic surface energies of InAs (a) (100), (b) (110), (c) (111)A, (d) (111)B, (e) (113)A, and (f) (113)B. When the vibrational energy was calculated, only the reconstructions represented by bold lines were considered. Note that Fig. S3(d) is identical to Fig. 5(b), except that there is no In vacancy α and β(2×2).

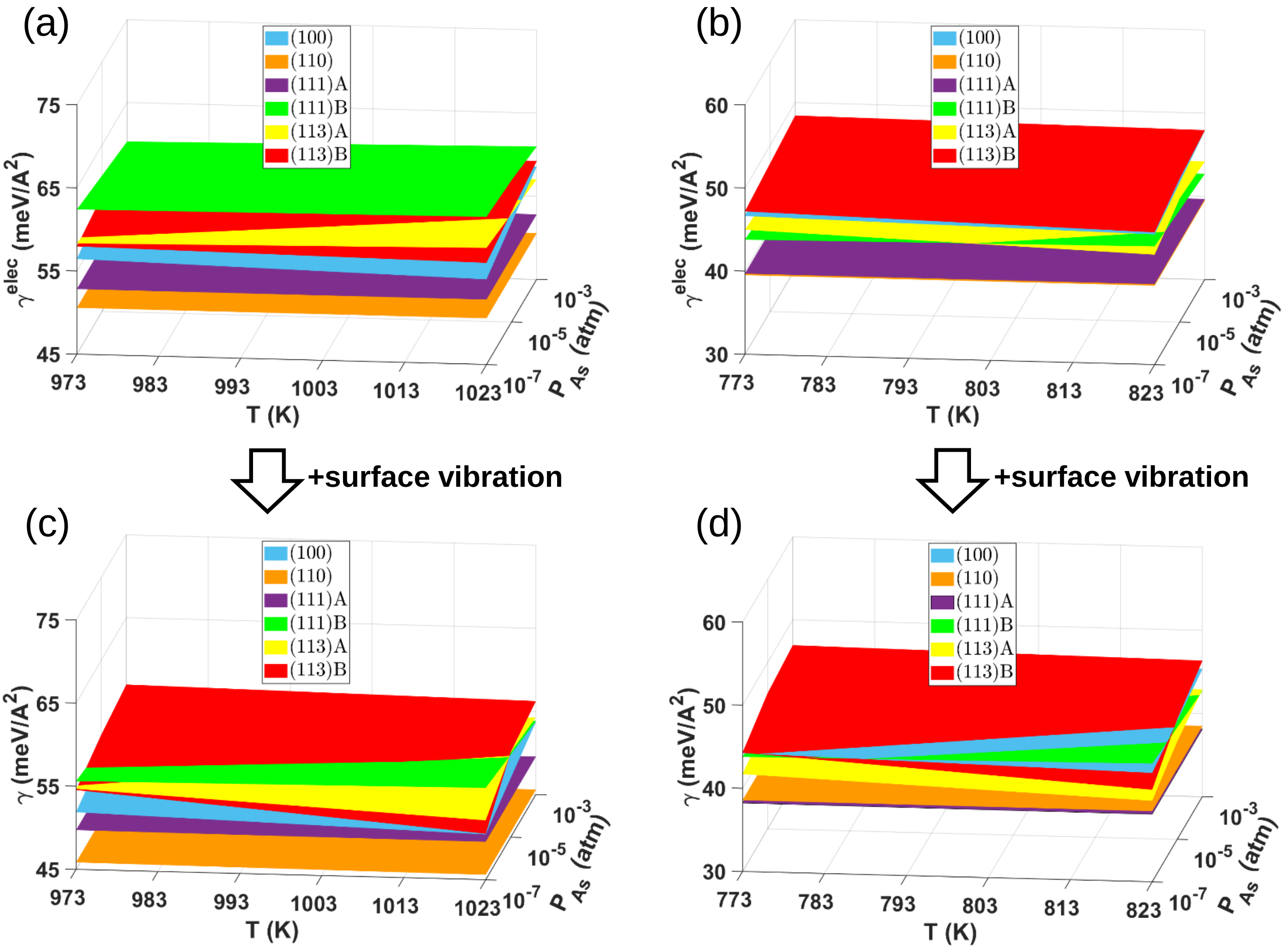

Figure S4. Calculated minimum surface energies (γ^{elec}) of (a) GaAs and (b) InAs in each surface orientation as a function of T and P_{As}. Minimum surface energy, including the electronic and vibrational energy terms ($\gamma = \gamma^{elec} + \Delta\gamma^{vib}$) of (c) GaAs and (d) InAs in each surface orientation as a function of T and P_{As} after considering the vibrational energy difference between the surface and the bulk ($\Delta\gamma^{vib}$).

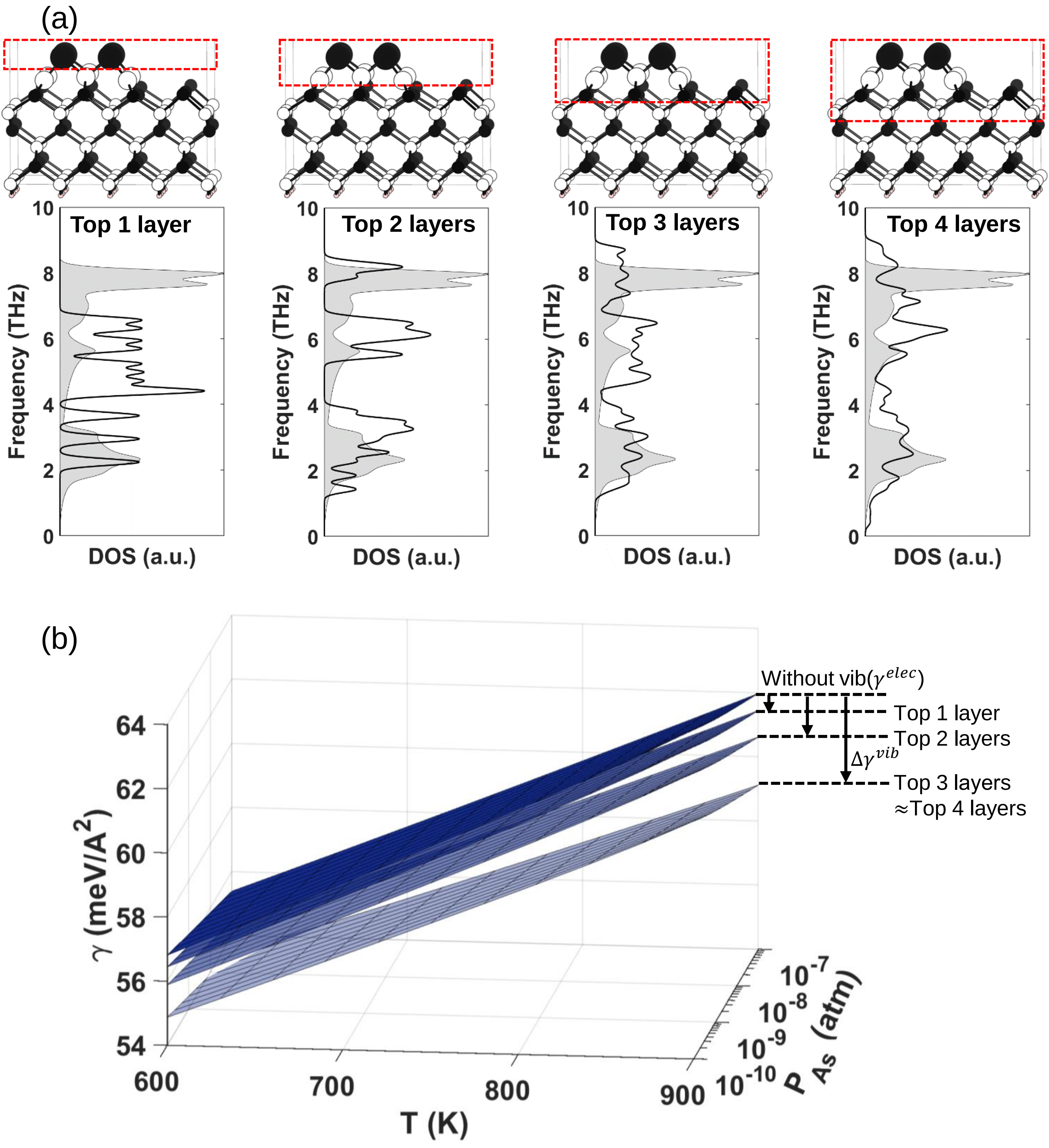

Figure S5. (a) Surface phonon DOS (black line) of the GaAs(100) β2(2×4) reconstruction after displacing the atoms at the uppermost layer, up to the 2nd layer, up to the 3rd layer, and up to the 4th layer from the uppermost layer, compared with the phonon DOS of the bulk GaAs (shaded area). (b) Surface energies composed of the electronic and vibrational terms ($\gamma = \gamma^{elec} + \Delta\gamma^{vib}$) of GaAs(100) β2(2×4).
